# F240. MULTI-MODAL PREDICTION OF GLOBAL FUNCTION FROM NEUROCOGNITIVE AND NEUROIMAGING MEASURES: OUTCOMES FROM THE PRONIA STUDY

**DOI:** 10.1093/schbul/sby017.771

**Published:** 2018-04-01

**Authors:** John Gillam, Dominic Dwyer, Anne Ruef, Nikolaos Koutsouleris, Stephen Wood

**Affiliations:** 1 University of Melbourne; 2 Ludwig-Maximilian-University; 3 Orygen, the National Centre of Excellence in Youth Mental Health

## Abstract

**Background:**

In order to extract the most powerful predictive models from data collected within the PRONIA study, diverse information sources must be combined. PRONIA aims to combine information from a range of study sites across Europe as well as from a diverse range of information sources. For each subject, neurocognitive, neuroimaging and clinically observed data has been collected that is intended to provide the basis for the development of predictive models for use in individualised diagnosis and prediction. However, as yet it is unclear as to which elements (or combination) of the measured data provide optimal predictive capacity and which features will generalize best.

**Methods:**

In order to combine data from a diverse range of sources a number of approaches may be considered. While it is initially attractive to concatenate the features gathered from each modality, this approach is problematic in two ways. Not only do the appropriate pre-processing steps differ between modalities, but the high dimensionality of imaging data (in comparison to neurocognitive measures) may alter the way each modality contributes to the decision function during learning. Instead, we investigate more simplistic learning approaches in an initial step that produces a single outcome for each modality considered. In a second step these outcomes are combined to generate a final estimate of the target class.

In this investigation neurocognitive and neuroimaging data, collected as part of the PRONIA study, were considered as features for prediction of clinically observed global function, measured at the same time-point. Each neurocognitive test, applied as part of the PRONIA battery, was considered as an independent modality, as were each of a range of MRI-based neuroimaging measures (from structural, functional and diffusion imaging). Support Vector Classification (SVC) was conducted for each modality, with the target class defined as a score of 65 or less on the Global Assessment of Function. Both linear classification and the use of radial basis functions were explored within the initial modality-independent learning phase as well as during modality fusion as part of the second learning phase. Repeated, nested, cross-validation was employed in both stages in order ensure robust estimates of generalisation.

**Results:**

Because each modality is reduced to a single measure in the first stage, each can contribute on an equal basis to the predictive outcome in the second while allowing inter-modality interaction. While SVC models do not naturally provide probabilistic outcomes, the distance of each point to the separating hyperplane can be scaled to represent the relative class probabilities. Predictions obtained at the first stage not only provide for the second phase of learning, but also provide a means to assess each modality for predictive accuracy. Correlations between the predictions from each mode provide information as to which combination of data may contribute constructively to the final outcome while learning approaches within the second phase can also be used to identify the most useful predictors.

**Discussion:**

The two-stage learning framework provides a useful approach to learning that allows assessment of each separate data stream as well as the fused-prediction outcome. The contribution of each data stream to the final prediction may be explored while interactions between data streams can also be contextualised. However, more subtle interactions between data, particularly at the initial input stage, may be difficult to observe and so the extension of this approach to more structured data-fusion and is considered.

